# The radioecological footprint of electricity production by wind turbines

**DOI:** 10.1093/rpd/ncad168

**Published:** 2023-06-23

**Authors:** Simon Bittner, Rainer Gellermann, Clemens Walther

**Affiliations:** Institute of Radioecology and Radiation Protection, Herrenhäuser Str. 2, Gebäude 4113, 30419 Hannover, Germany; Nuclear Control & Consulting GmbH, Hinter dem Turme 24, 38114 Braunschweig, Germany; Institute of Radioecology and Radiation Protection, Herrenhäuser Str. 2, Gebäude 4113, 30419 Hannover, Germany

## Abstract

The worldwide transformation of electricity production goes hand in hand with increasing use of wind energy. The German ‘Energiewende’ project is no exception and relies heavily on the construction and use of an ever-increasing number of wind turbines. While the operation of wind turbines does not lead to the emission of pollutants (in contrast to, e.g. coal, oil or gas), the production processes of the construction materials do. Since the raw materials’ production primarily takes place outside Germany, radioactivity and doses related to these processes occur at remote places in the world. This effect might be called an ‘export of doses’. In the present paper, we perform a life cycle analysis of wind turbines, investigating the mining and production of the construction materials. We focus on rare-earth elements needed for the generator magnets and assess the associated releases of radioactive materials during mining and processing, primarily in China. Estimates of dose to the public in selected Chinese cities are calculated. Different electricity generation techniques are compared by the use of the quantity (collective) dose per GW per year.

## Introduction

As part of the so-called ‘Energiewende’, Germany started the process of transforming the national electricity production. Conventional large-scale power plants using coal, lignite, oil or uranium for fuel are being replaced by decentralised renewable technologies^(1)^. Also, on a global scale, the installed capacity of photovoltaic plants and wind turbines increases continuously. The global primary energy produced from renewable sources increased by +3.2 EJ in 2019. The largest contributor with an increase of +1.4 EJ is the energy production because of wind, followed by solar with +1.2 EJ^(2)^.

The increasing share of renewables fosters the need for evaluating potential risks to man and environment. The present work examines in an exemplary approach the radioecological footprint of wind turbine production and operation in a life cycle analysis. The results help to identify high exposure situations and may help to put the radioactivity in wind turbines in a broader perspective. Furthermore, awareness needs to be raised on how low-emission technologies in one country might lead to radiation doses and risk to workers and the population in others. While this ‘export of doses’ is a very general issue in today’s globalised economy, the present work focuses on radiological risk arising from the mining of rare-earth minerals for the production of neodymium required for building a specific type of wind turbine generator.

From the literature data of selected mining and production areas, doses to workers and the general population are estimated. These doses are normalised to unit electricity produced by wind turbines and are compared with values for electricity production by conventional plants. Finally, the collective dose per GW per year is compared with the estimated values of the UNSCEAR report from 2016^(3)^.

## Method of this study

The study relies on desk research with special focus on data from peer-reviewed articles and conference papers. To cover the production chain of the construction materials several production steps are investigated. However, the literature for those steps is scarce. Only little additional literature to check the reliability is published. For the raw data of the production steps, only publications indexed in the Web of Science are used, to ensure a minimum quality.

As a preliminary working hypothesis, we assume that the limited data available is representative of the examined processing step. Generally, many numbers suffer from a large uncertainty, which is accounted for in the model calculations done. In order to perform weighted parameter fits, assumptions concerning the weighting factors were made and detailed at the respective place in the text. It should be considered that the dose values obtained in this work result mainly from conservative assumptions.

To calculate the received doses because of the use of wind energy for electricity generation, a life cycle analysis of the wind turbines is performed using the following assumptions: Only the mining and processing of raw materials needed for construction of wind turbines leads to significant exposure. Exposures during erection, operation, decommissioning and afterwards are not considered significant. Furthermore, maintenance (e.g. generator replacement) and material recycling are not considered.

For the examination, a direct drive 3 MW wind turbine with a lifetime of 22.5 y and an efficiency of 24% is chosen. For this type of turbine, information about the constituents and materials contained is available. The average turbine capacity, considering all operating turbines, in Germany amounts to 2 MW^(4)^ today. However, more and more large turbines are built up to 5 MW capacity^(5)^, indicating an increase in the average size in the near future, which motivates our choice of 3 MW for the present calculation. In 2019, 26% of all turbines worldwide were equipped with direct drive generators^(6)^. Ault *et al*.^(7)^ estimate that in 80% of direct drive generators, rare-earth magnets are used. This usage of rare-earth elements causes particularly high radiological consequences (as we show later). Therefore, we consider as an example the direct-drive generator with a neodymium magnet for our calculations. A 22.5 y lifetime is chosen, motivated by the interval ranging from 20 to 25 y given by Jensen *et al*.^(8)^. The mean efficiency of 24% is estimated from the mean electricity production data of 2020 in Germany^(9)^.

Jensen *et al*.^(8)^ examined the total material requirements of a wind farm. They investigated the recycling potential of a 60 MW wind farm and listed the amount of material needed. No information on the number of turbines is given. However, the fact that direct-drive 3 MW turbines are discussed in detail, and neodymium magnets are considered motivates the assumed partition of 20 wind turbines of 3 MW each.

**Table 1:** Total used materials for a 60 MW wind farm from Jensen *et al*.^(8)^.

Material	Mass (kg)
Ferrous metal	6 560 000
Aluminium	104 000
Composite materials	660 000
Lubricating oil	30 000
Electronics	124 000
Batteries	36 000
Fluorescent lamps	3 800
NdFeB magnet	40 000
Copper	292 000
Balsa Wood	29 000
Plastic	44 600
Total	7 923 400

Table 1 compiles the masses of the materials based on the numbers provided by Jensen *et al*. Ferrous metals and composite material account for the biggest share. Suffice data for examining the radioactivity in different parts of the production chain of these materials are available only for ferrous metals and neodymium. From these two, neodymium is, according to our estimations below, far more relevant from the radiological point of view.

### Dose calculation

Our analysis is focused on the exposure related to the mining and processing of rare-earth minerals. Neodymium itself has a very long-lived radionuclide ^144^Nd (${T}_{1/2}=2.2\cdot{10}^{15}\mathrm{a}$, alpha decay), but its radioactivity can be disregarded from the radiation protection point of view. Other rare-earth elements also contain radioactive isotopes with a very large half-life (e.g. ^138^La, ^147^Sm and ^176^Lu) that do not really contribute to doses.

Relevant exposure originates from uranium and thorium present in the rare-earth minerals^(7)^. Even though data of decay-series radionuclides in metallic neodymium have not yet been published in the available literature, the processing and purification of the neodymium^(10)^ remove uranium and thorium as well as their decay products, which motivate our assumption that the magnets do not contain relevant amounts of radioactivity. Consequently, exposure during operation, construction, decommissioning of wind turbines and disposal of waste on landfills is negligible from a radiation protection point of view.

From the data of Jensen *et al*.^(8)^ for the 60 MW wind farm, we calculate 328 000 kg ferrous metals required for the structural material of one 3 MW turbine. Its magnet weight is calculated to be 2000 kg, from which ~72 wt% is attributed to iron^(11)^, resulting in the used values of 560 kg for neodymium and 1440 kg for iron. We do not modify the amount of needed material in between production steps, e.g. to account for ore grades.

In order to obtain estimates of annual collective dose, we assess the production processes of iron and neodymium, respectively. In the case of neodymium mining, two major parameters need to be taken into account. We start with data for aqueous and/or gaseous discharge of the mine or production facility. Besides neodymium, often other elements are mined and processed. Hence, we have to calculate the proportion of neodymium of the total production and take into account only this fraction for calculating dose. If, for instance, neodymium accounts for 20% of the total mass, 20% of the annual effective dose because of the facilities’ release will be included in our calculation for the production of generator magnets. In a second step, we calculate the fraction of 1-y production quantity that is needed to build the magnet of one 3 MW turbine generator.

Since all available data do not allow a comprehensive statistical analysis of the collective effective dose, we estimate ranges from the upper and lower boundaries of the input parameters. This may lead to asymmetric intervals. Because of a lack of data, in some cases, different assumptions were made, noted at the respective text passage.

The calculation and the used values for the parameters listed in Table 2 are similar to those proposed in the UNSCEAR 2000 report^(12)^. Each step of the production process is discussed separately, and the parameter values are stated. The values taken from the literature are based on different types of measurements. Consequently, the mathematical model for calculating the additional annual effective dose depends on the specific set of data; however, particular attention is paid to its compliance with the UNSCEAR 2000 report. The following calculation is unified for every data set.

**Table 2:** List of general parameters.

Symbol	Name	Value	Unit
S	Collective dose		manSv
S_a_	Sum of collective dose per GW per year		manSv GW^−1^ a^−1^
E	Additional annual effective dose		mSv
E^*^	Weighted additional annual effective dose		mSv
N	Number of persons who were exposed		
m	Amount of material needed for 3 MW wind turbine		kg
M	Annual amount of produced material		kg
P	Installed power of the wind turbine	0.003	GW
$\eta$	Efficiency	0.24	
$\tau$	Lifetime of the wind turbine	22.5	a

From the annual effective dose *E* per member of the public or per worker because of operation of the mine or plant, the ‘weighted additional annual effective dose’ E^*^ because of the production of material for one 3 MW turbine is calculated as follows: 


(1) \begin{equation*} {\mathrm{E}}^{\ast }=\frac{\mathrm{E}}{\mathrm{M}}\cdot \mathrm{m}. \end{equation*}

*M* denotes the total annual amount of mined or produced material and *m* the material needed for one 3 MW wind turbine. Multiplying E^*^ by the number of exposed people (*N*) results in the collective dose (*S*):


(2) \begin{equation*} \mathrm{S}={\mathrm{E}}^{\ast}\cdot \mathrm{N}. \end{equation*}

Finally, the sum of collective doses because of the production of both materials is normalised to the amount of electric energy produced during the lifetime of the wind turbine. This normalisation was adapted from the UNSCEAR 2016 report^(3)^ for the sake of comparability.

This normalised collective dose *S_a_* is referred to the produced energy, calculated by: 


(3) \begin{equation*} {\mathrm{S}}_{\mathrm{a}}=\frac{\sum_{\mathrm i}{\mathrm S}_{\mathrm i}}{\mathrm{P}\cdot \mathrm{\eta} \cdot \mathrm{\tau}\ } \end{equation*}

where *τ* is the lifetime of the turbine, *η* the mean efficiency for electricity production (produced energy/installed capacity × time) and *P* the installed capacity.

In this study, we consider only two different materials, ferrous materials and neodymium. It must be mentioned that the production of other materials in Table 1 is connected with NORM and, consequently, with radiation exposures, too. For instance, the oil and gas production, the basis of organic compounds, the mining of ores other than uranium (like copper) and the production of tin, copper, aluminium, zinc, lead, and iron and steel are listed in the IAEA report^(13)^ as industry sectors are most likely to require regulatory consideration from the radiation protection point of view.

**Table 3:** Raw data used for the dose estimation attributed to ferrous metals.

	^226^Ra	^232^Th	^40^K	Unit	Reference
Tailing pond	24 (17–32)	14 (9.3–18)	738 (717–762)	Bq kg^−1^	^(14)^
Tailing pond background	32	41	440	Bq kg^−1^	^(12)^
	^**210**^ **Pb**	^**210**^ **Po**			
Dust of blast furnace	2340 (2304–2376)	470 (455–485)		Bq kg^−1^	^(21)^
Airborne conc around steel plant	812 (618–1087)	291 (154–564)		${\mu}$ Bq m^−3^	^(16)^
Around steel plant background	399 (382–416)	48.5 (45.2–51.8)		${\mu}$ Bq m^−3^	^(16)^

### Ferrous metals

First, the exposure because of the tailing of iron ore mining is examined^(8, 12,14–18)^. Zhuang *et al*.^(14)^ measured the nuclides ^40^K, ^226^Ra and ^232^Th on the tailing. We assume that workers of that facility are exposed to external gamma rays while working on the tailing pond.

Second, the exposure of persons of the public as a consequence of the production of steel is considered. The main exposure source in processes related to steel production is dust released from the blast furnace process. This dust is characterised by enhanced ^210^Pb and ^210^Po activities (M5-material according to Gellermann *et al.*^(19)^).

Jia *et al*.^(16)^ measured ^210^Pb and ^210^Po in the vicinity of a steel plant. An estimate of the effective dose because of ingestion via the dry deposition pathway on plant-based products (in compliance with^(20)^) yields ~500 ${\mu}$Sv per year for infants. If one assumes that a share of 10% of the consumed plants is grown locally and excludes infants, then the remaining dose is small. Therefore, we only include the path of inhalation for this source, because of fewer presumptions. Certainly, those arguments are heavily dependent on the respective region.

Another set of data relevant to workers was measured inside a steel plant by Khater and Bakr^(21)^. The obtained concentrations of ^210^Po and ^210^Pb in a sample of blast furnace dust are used in this work to calculate the exposure resulting from inhalation for workers^(12, 17, 18)^. All data sets related to ferrous metals are listed again in Table 3. The dose coefficient related to dust inhalation depends on the ratio between ^210^Pb and ^210^Po. With the activity concentrations given in Table 3, the effective dose coefficient is about 2.5 ${\mu}$Sv/Bq ^210^Pb.

### Neodymium

For neodymium, the available data were mainly measured around the region of Bayan Obo. This is a Chinese iron mine, which outputs rare-earth metals as a by-product^(7)^. As the main producer, China dominates the rare-earth metals market^(7, 22)^. Though only from a single mine, the data may nevertheless be considered representative, since it is the world’s largest rare-earth deposit. More than 80% of the Chinese light rare-earth resources are located there ^(23)^. The mine is located in the administrative area of Baotou in the northern part of China. The smelting of the ore takes place in the next biggest city Baotou.

### Dose because of inhalation in the mining area and in the smelting area

The radiological situation because of inhalation exposure around the Chinese mining and smelting districts was examined by Wang *et al*.^(24)^. The samples were obtained by a personal inhalation exposure sampler (cut size $10\ {\mu} \mathrm{m}$), which was worn by 10 volunteers near the mining area (Bayan Obo) and by nine volunteers near the smelting area (Baotou City). The samplers were worn for 24 h, and each volunteer did wear three samplers on consecutive days. The activity concentration of ^232^Th (${\mathrm{C}}_{\mathrm{Th}-232}$) was examined.

**Table 4:** Concentrations in the samplers conducted by Wang *et al*.^(24)^.

	Bayan Obo ($\mathrm{mBq}\ {\mathrm{m}}^{-3}$)	Baotou City $\left(\mathrm{mBq}\ {\mathrm{m}}^{-3}\right)$
^ 232^Th	5.97 (1.81–11.62)	5.79 (2.38–11.82)

A mean ^232^Th-concentration of 5.97 mBq m^−3^ for the mining area and 5.79 mBq m^−3^ for the smelting area was found ^(24)^, again listed in Table 4. This is much more than the natural background activity, which can be estimated from a mean dust concentration of 0.05–0.1 mg/m^3^ and a ^232^Th-activity concentration in soil of 0.02–0.05 Bq/g to be about 0.001–0.005 mBq m^−3^. This range of background concentrations is orders of magnitude lower than the measured ^232^Th-concentrations in the mining and smelting areas, which justifies neglecting the background. Because dust from mining comes from minerals that have been treated by mechanical processes only, it can be classified as ‘M1-material’ according to Gellermann *et al*.^(19)^, and the ^232^Th-decay chain has to be considered in secular equilibrium.

**Table 5:** Calculation of dose coefficient for inhalation of minerals of neodymium production (N-processes) containing ^232^Th, ^238^U and ^235^U series. Occupational coefficients are taken from ICRP 119^(26)^. The weights indicated by ^a^ are calculated by scaling the activity of ^238^U to ^235^U (${\mathrm{w}}_{\mathrm{235}\mathrm{U}}={\mathrm{w}}_{\mathrm{238}\mathrm{U}}/{\mathrm{A}}_{\mathrm{s}\mathrm{238}\mathrm{U}} \cdot \mathrm{f} \cdot{\mathrm{A}}_{\mathrm{s}\mathrm{235}\mathrm{U}}$). Here *w* denotes the weights, *A*_*s*_ the specific activity and *f* (=0.007204) refers to the relative abundance of ^235^U.

Nuclid-specific dose coefficient	Value (${\mu}$Sv Bq^−1^)	Weight	Weighted value (${\mu}$Sv Bq^−1^)
^232^Th (slow, 5 ${\mu}\! {\textrm{m}}$)	12	1	12
^228^Ra (medium, 5 ${\mu}\! \mathrm{m}$)	1.7	1	1.7
^228^Th (slow, 5 ${\mu}\! \mathrm{m}$)	25	1	25
^224^Ra (medium, 5 ${\mu} \! \mathrm{m}$)	2.4	1	2.4
^238^U (medium, 5 ${\mu} \! \mathrm{m}$)	1.6	0.25	0.4
^234^U (medium, 5 ${\mu} \mathrm{m}$)	2.1	0.25	0.525
^230^Th (slow, 5 ${\mu} \! \mathrm{m}$)	7.2	0.25	1.8
^226^Ra (medium, 5 ${\mu} \! \mathrm{m}$)	2.2	0.25	0.55
^210^Pb (fast, 5 ${\mu} \! \mathrm{m}$)	1.1	0.25	0.275
^210^Po (medium, 5 ${\mu} \! \mathrm{m}$)	2.2	0.25	0.55
^235^U (medium, 5 ${\mu} \! \mathrm{m}$)	1.8	0.0116^a^	0.02
^231^Pa (medium, 5 ${\mu} \! \mathrm{m}$)	89	0.0116^a^	1.03
^227^Ac (fast, 5 ${\mu} \! \mathrm{m}$)	630	0.0116^a^	7.31
Total (per Bq ^232^Th)			53.56

The following model of exposure is used to determine the annual effective dose because of inhalation of dust: 


(4) \begin{equation*} \mathrm{E}={\mathrm{C}}_{\mathrm{Th}-232}\cdot \mathrm{B}\cdot \mathrm{d}\cdot \left(\left(1-\mathrm{F}\ \right)+ {\mathrm{F}}_{\mathrm r}\cdot \mathrm{F}\ \right) \end{equation*}

*B* is the breathing rate, *F* the indoor occupancy factor and *F_r_* denotes the indoor air to outdoor air concentration ratio of ^232^Th. According to data given in the Safety Report No. 68 of the IAEA^(25)^, the activity ratio ^238^U/^232^Th can be estimated of about 0.1–0.25. With that information, we calculate a combined effective dose coefficient *d* for ^232^Th, ^238^U and ^235^U series and their decay products to be 53.56 ${\mu}$Sv/Bq ^232^Th (see Table 5 for details). This is several times higher than the dose coefficient of dust from the steel production^(19)^. Because the sampler used for the aerosol collection had a cut-off of 10 ${\mu}$m, dose coefficients according to ICRP 119 for members of the public that are defined for aerosols with AMAD 1 ${\mu}$m overestimate the doses. For that reason, we apply dose coefficients for aerosols of AMAD 5 ${\mu}$m for occupational exposure from ICRP 119 for dose estimations of persons of the public, too. Furthermore, we use the dose coefficients for lung-clearance class M except for thorium isotopes (Class S) and Ac-227 (Class F) ^(27)^.

Adapting all values from the UNSCEAR report^(12)^, we used the breathing rate of an adult person of the public *B* = $22.2\ {\mathrm{m}}^3{\mathrm{d}}^{-1}$, the indoor occupancy factor *F* = 0.8 (contrary to 0.92 in the original work ^(24)^) and *F_r_* = 0.3. The parameters are listed again in Table 6. The next equation shows an example calculation for the mining area: 


(5) \begin{align*} {\mathrm{E}} = \ & 5.97\cdot{10}^{-3}\ \mathrm{Bq}\ {\mathrm{m}}^{-3}\cdot{}22.2\ {\mathrm{m}}^3{\mathrm{d}}^{-1}\cdot 365\ \mathrm{d}\quad \notag\\[5pt]&\cdot 0.05356\ \mathrm{mSv}\ {\mathrm{Bq}}^{-1}\cdot ((1-0.8) +0.8 \cdot 0.3) \\[5pt]= \ &1.14\ \mathrm{mSv}. \notag \end{align*}

**Table 6:** Parameters and values for the calculation of the inhalation in the mining area and in the smelting area, ^a^ indicates a particular value of the raw data.

Symbol	Name	Value	Unit
E	Additional annual effective dose		mSv
B	Breathing rate for persons of the public	22.2	m^3^ d^−1^
F	Indoor occupancy factor	0.8	
F_r_	Ratio of indoor to outdoor air concentration	0.3	
d	Combined effective dose coefficient for the ^232^Th, ^238^U and ^235^U series (see Table 5)	0.05356	$\mathrm{m}$ Sv Bq^−1^
${\mathrm C}_{\mathrm{Th-232}}$	Activity concentration in samplers	5.97^a^	mBq m^−3^
m	Needed mass of neodymium per 3 MW turbine	560	kg
M_mine_	Total mass of produced rare-earth metals	55 000 000	kg
M_smelting_	Total mass of produced rare-earth metals	55 000 000	kg
${\mathrm N}_{\mathrm{mine}}^{\mathrm{living}}$	Number of exposed inhabitants in Bayan Obo	23 000	
${\mathrm N}_{\mathrm{smelting}}^{\mathrm{living}}$	Number of exposed inhabitants in Baotou City	1 510 000	

Population numbers are taken from a web-based source, referring to the 2020 Chinese census^(28)^. Bayan Obo is listed with 23 000 inhabitants. Baotou City, on the other hand, consists of multiple districts. After comparing the sampling locations from Li *et al*.^(29)^ (discussed in the next chapter) with the districts, we consider inhabitants of Kūndūlún Qū (790 000) and Qīngshān Qū (720 000) to be exposed to the airborne ^232^Th concentrations. This approach leads to identical population numbers for this and the following sources.

The mine produces 55 000 metric tons of rare-earth metals per year ^(7)^. We assume that the full production of the mine is smelted in the metallurgical area in the western part of the city Baotou because the two areas (~150 km apart) are connected by a railway, which is used 24 h each day for the transport of ore. This information is taken from an environmental impact study of that transport by Wang *et al*.^(30)^.

With these values for population and production, the weighted effective dose and the collective dose are calculated, here for Bayan Obo: 


(6) \begin{equation*} {\displaystyle \begin{array}{l} {\mathrm{E}}^\ast = 1.14\cdot{10}^{-3}\ \mathrm{Sv}\cdot \displaystyle\frac{560\ \mathrm{kg}}{55\ 000\ 000\ \mathrm{kg}}\\[10pt] {}\quad =1.16\cdot{10}^{-8}\ \mathrm{Sv}\ \ \end{array} } \end{equation*}

(7) \begin{equation*} {\displaystyle \begin{array}{l} \ \ \ {\mathrm{S}} = 1.16\cdot{10}^{-8}\ \mathrm{Sv}\cdot 23\ 000 \quad\quad \, \\[7pt] \ \ \, \quad= 2.67\cdot{10}^{-4}\ \mathrm{manSv}. \end{array}\quad}\quad \end{equation*}

**Table 7:** Raw data from Li *et al*. for the mining area (Bayan Obo) and the smelting area (Baotou City)^(29)^. The values represent the share of mass of the element in the examined sample. The values of potassium are converted from % to mg/kg in comparison to the original data.

	Tailing (mg kg^−1^)	Next to tailing (mg kg^−1^)	Living area (mg kg^−1^)
Thorium, background 9.30 mg kg^−1^			
Bayan Obo	276 (275–276)	39.6 (2.40–81.6)	26.2 (17.1–35.2)
Baotou City	321 (290–352)	13.4 (8.74–18.1)	12.6 (7.80–17.4)
Uranium, background 2.55 mg kg^−1^			
Bayan Obo	5.70 (5.13–6.27)	1.92 (1.34–2.50)	2.93 (2.78–3.08)
Baotou City	3.02 (1.98–4.06)	2.22 (1.85–2.59)	2.88 (1.24–4.52)
Potassium, background 21 800 mg kg^−1^			
Bayan Obo	20 500 (19 800–21 200)	30 100 (25 000–35 200)	31 300 (28 800–33 800)
Baotou City	28 600 (25 900–31 300)	19 000 (15 600–22 400)	22 400 (14 900–29 900)

### Dose because of external gamma-rays in the mining area and in the smelting area

The concentration of nuclides in soil was examined with in situ gamma-ray spectrometry by Li *et al*.^(29)^. Sampling took place in the mining area as well as in the smelting area. For both areas, the samples are categorised into:

(1) taken from the living area
(2) next to the tailing dam
(3) directly from the tailing.

For every category, the content of the elements potassium, uranium and thorium (${F}_i$) was examined. The original data including the background is listed in Table 7. 


(8) \begin{equation*} {\mathrm{E}}_{\mathrm{i}}=\left(\left({\mathrm{F}}_{\mathrm{i}}-{\mathrm{F}}_{\mathrm i}^{\ast}\right)\cdot{\mathrm{f}}_{\mathrm{i}}\cdot{\mathrm{A}}_{\mathrm{si}}\right)\cdot{\mathrm{d}}_{\mathrm{i}} \end{equation*}

(9) \begin{equation*} \mathrm{E}=\left({\mathrm{E}}_{\mathrm{K}}+{\mathrm{E}}_{\mathrm{Th}}+{\mathrm{E}}_{\mathrm{U}}\right)\cdot \mathrm{t}. \end{equation*}

The annual effective dose because of external exposure by gamma rays from ^40^K, ^232^Th and ^238^U is obtained by using:

Here ${F}_i^{\ast }$ denotes the measured background from Li *et al*.^(29)^. ${d}_i$ are the dose coefficients from the UNSCEAR report of the year 2000^(12)^, ${f}_i$ are the relative abundances of the elements’ isotopes and the specific activities ${A}_{si}$ are calculated from Vogt and Vahlbruch^(31)^. The parameters are found in Table 8. The dose coefficients account for effective dose from the ^232^Th and ^238^U series by assuming an equilibrium and a thick contaminated soil. Because the excess activity in the living areas comes from the dust deposition, the thickness of the contamination is estimated to be smaller than 0.2 m based on experience with depth distributions of ^137^Cs or ^210^Pb in soil ^(32)^. Therefore, the application of the dose coefficient of thick contaminated layers results in a conservative value.

**Table 8:** Parameters and values for the calculation of dose because of external gamma-rays in the mining area and in the smelting area, ^a^ indicates a particular value of the raw data.

Symbol	Name	Value	Unit
E	Additional annual effective dose		mSv
${\mathrm d}_{\mathrm K}$	Dose coefficient for external gamma because of NORM for ^40^K	0.029	nSv kg h^−1^ Bq^−1^
${\mathrm d}_{\mathrm{Th}}$	Dose coefficient for external gamma because of NORM for ^232^Th-decay series	0.46	nSv kg h^−1^ Bq^−1^
${\mathrm d}_{\mathrm U}$	Dose coefficient for external gamma because of NORM for ^238^U-decay series	0.30	nSv kg h^−1^ Bq^−1^
${\mathrm f}_{\mathrm K}$	Natural mass share of ^40^K from potassium	0.000117	
${\mathrm f}_{\mathrm{Th}}$	Natural mass share of ^232^Th from thorium	1	
${\mathrm f}_{\mathrm U}$	Natural mass share of ^238^U from uranium	0.9927	
${\mathrm F}_{\mathrm K}$	Share of the potassium in the ground	31 300^a^	mg kg^−1^
${\mathrm F}_{\mathrm{Th}}$	Share of the thorium in the ground	26.2^a^	mg kg^−1^
${\mathrm F}_{\mathrm U}$	Share of the uranium in the ground	2.93^a^	mg kg^−1^
${\mathrm A}_{\mathrm{sK}}$	Specific activity ^40^K	265	Bq mg^−1^
${\mathrm A}_{\mathrm{sTh}}$	Specific activity ^232^Th	4.05	Bq mg^−1^
${\mathrm A}_{\mathrm{sU}}$	Specific activity ^238^U	12.4	Bq mg^−1^
t_only_tailing_	Annual duration of exposure on tailing	500	h
t_work_	Annual duration of exposure next to tailing	2000	h
t_living_	Annual duration of exposure while in living area	8760	h
m	Needed mass of neodymium per 3 MW turbine	560	kg
${\mathrm M}_{\mathrm{mine}}$	Total mass of produced rare-earth metals	55 000 000	kg
${\mathrm M}_{\mathrm{smelting}}$	Total mass of produced rare-earth metals	55 000 000	kg
${\mathrm N}_{\mathrm{mine}}^{\mathrm{tailing}}$	Number of exposed workers working on the tailing (Bayan Obo)	100	
${\mathrm N}_{\mathrm{mine}}^{\mathrm{work}}$	Number of exposed workers working next to tailing (Bayan Obo)	6000–100	
${\mathrm N}_{\mathrm{mine}}^{\mathrm{living}}$	Number of exposed inhabitants in living area (Bayan Obo)	23 000–6000	
${\mathrm N}_{\mathrm{smelting}}^{\mathrm{tailing}}$	Number of exposed workers working on the tailing (Baotou City)	600	
${\mathrm N}_{\mathrm{smelting}}^{\mathrm{work}}$	Number of exposed workers working next to tailing (Baotou City)	40 000–600	
${\mathrm N}_{\mathrm{smelting}}^{\mathrm{living}}$	Number of exposed inhabitants in living area (Baotou City)	1 510 000–40 000	

An example calculation for the mining area is as follows: 


(10) \begin{align*} \displaystyle \begin{array}{l} {\mathrm{E}}_{\mathrm{living}}\! =\! \left[\!\left(9500\ \mathrm{m}\mathrm{g}\ {\mathrm{kg}}^{-1}\cdot 1.17\cdot{10}^{-4} \cdot 265\ \mathrm{B}\mathrm{q}\ {\mathrm{mg}}^{-1}\!\right)\right.\\[10pt] \qquad\qquad\cdot 0.029\cdot{10}^{-9}\ \mathrm{Sv}\ \mathrm{k}\mathrm{g}\ {\mathrm{h}}^{-1}\ \mathrm{B}{\mathrm{q}}^{-1}\\[10pt] \qquad\qquad + \left(0.38\ \mathrm{m}\mathrm{g}\ \mathrm{k}{\mathrm{g}}^{-1}\cdot 0.9927\cdot 12.4\ \mathrm{B}\mathrm{q}\ \mathrm{m}{\mathrm{g}}^{-1}\right)\\[10pt] \qquad\qquad\cdot 0.3\cdot{10}^{-9}\ \mathrm{Sv}\ \mathrm{k}\mathrm{g}\ {\mathrm{h}}^{-1}\ \mathrm{B}{\mathrm{q}}^{-1}\\[10pt] \qquad\qquad +\left(16.9\ \mathrm{m}\mathrm{g}\ \mathrm{k}{\mathrm{g}}^{-1}\cdot 1\cdot 4.05\ \mathrm{B}\mathrm{q}\ \mathrm{m}{\mathrm{g}}^{-1}\right)\\[10pt] \qquad\qquad\left.\cdot 0.46\cdot{10}^{-9}\ \mathrm{Sv}\ \mathrm{k}\mathrm{g}\ {\mathrm{h}}^{-1}\ \mathrm{B}{\mathrm{q}}^{-1}\right]\cdot{10}^3\cdot 8\ 760\ \mathrm{h}. \\[10pt] \qquad \ \, \ =0.363\ \mathrm{m}\mathrm{Sv.} \end{array} \end{align*}

The categorisation of Li *et al*.^(29)^ enables the distinction between three different exposure scenarios for the representative person.

(1) Resident in the living area
(2) Worker at the facility
(3) Worker on top of the tailing

In 1 year (8760 h), the residents obtain an additional dose of *E_living_.* The occupational dose of workers, however, depends on the location of their workplace. Workers not employed on the tailing pond receive *E_work_*, which assumes 2000 h per year at the facility (50 weeks per year, 5 d per week). For persons who are workings on the tailing pond, we assume a schedule of 2 h per day (500 h per year) on the tailing pond, adapted from Liu and Pan^(15)^, and the remaining 6 h (1500 h per year) at the facility. This approach yields *E_work,tailing_*, which is calculated by adding the dose received on the tailing (*E_only_tailing_*) and the dose, scaled to the exposure time, from other work within the facility: 


(11) \begin{equation*} {\mathrm{E}}_{\mathrm{work},\mathrm{tailing}}={\mathrm{E}}_{\mathrm{only}\_\mathrm{tailing}}+\frac{1\ 500\ \mathrm{h}}{2\ 000\ \mathrm{h}}\cdot{\mathrm{E}}_{\mathrm{work}}. \end{equation*}

Following the argument of the previous chapter, we assume 23 000 residents for Bayan Obo, the town close to the mining area. The two districts Kūndūlún Qū and Qīngshān Qū are used as an estimate for the number of exposed people in Baotou City because they are the closest to the facility and match with the location of measurements by Li *et al*.^(29)^. As stated by Ault *et al*.^(7)^, 6000 workers are employed in the mine at Bayan Obo. Assuming the same fraction of the population to be employed in facilities for Baotou City as in Bayan Obo would overestimate the exposed workforce as there should be a larger secondary sector in the big city. We limit the number of workers to 10% of that fraction, see Equation 12, which yields ~40 000 exposed workers. No data are available to quantify the number of workers on the tailing for the last category. We assume 100 workers for Bayan Obo and 600 for Baotou City. 


(12) \begin{equation*} {\mathrm N}_{\mathrm{smelting}}^{\mathrm{work}}=\frac{{\mathrm{N}}_{\mathrm{mine}}^{\mathrm{work}}}{\mathrm{N}_{\mathrm{mine}}^{\mathrm{living}}}\cdot{\mathrm N}_{\mathrm{smelting}}^{\mathrm{living}} \cdot 0.1 \end{equation*}

**Table 9:** Raw data for the processing plant from Haridasan *et al*.^(33)^.

Type	Value	Unit
External gamma exposure	0.50 (0.30–0.80)	${\mu} \mathrm{Gy}\ {\mathrm{h}}^{-1}$
External gamma exposure background	0.15	${\mu} \mathrm{Gy}\ {\mathrm{h}}^{-1}$
^220^Rn progeny	41 (31–51)	$\mathrm{mWL}\ {\mathrm{a}}^{-1}$
Airborne ^232^Th	3 (2–4)	$\mathrm{mBq}\ {\mathrm{m}}^{-3}$

A person working in the mine most likely resides in the city. To avoid double counting a person, we deduct the number of workers from the residents. The calculation of the weighted annual effective dose and the collective dose for members of the public living in the mining area is performed according to the following equations:


(13) \begin{equation*}\begin{array}{l}\displaystyle{\mathrm E}^{\ast} =0.363 \cdot 10^{-3} \ \mathrm{Sv} \cdot \frac{560\ \mathrm{kg}}{55\ 000\ 000\ \mathrm{kg}}\\ \quad \ \, =3.70 \cdot 10^{-9} \ \mathrm{Sv}\end{array}\end{equation*}

(14) \begin{equation*} \begin{array}{l} \ \ {\mathrm S} = 3.70\cdot{10}^{-9}\ \mathrm{Sv}\cdot \left(23\ 000-6000\right) \\ \quad \, \, =6.29\cdot{10}^{-5} \ \mathrm{manSv} \end{array}\quad\quad \end{equation*}

### Exposure because of the processing of rare-earths

The exposure of employees working in a processing plant for rare-earth minerals located in Kerala, India, was examined by Haridasan *et al*.^(33)^. The external gamma exposure was measured monthly with a scintillometer at different locations in the facility. In addition, air samples were collected, which were used to estimate the potential alpha energy concentration (PAEC) because of ^220^Rn progeny and the concentration of long-lived alpha-emitting nuclides. By assuming equilibrium up to ^224^Ra, Haridasan *et al*.^(33)^ determined a so-called equivalent activity of ^232^Th (${C}_{Th}$), see Table 9.

The following model is used to calculate the inhalation dose for the employees because of the equivalent activity of ^232^Th assuming equilibrium up to ^224^Ra: 


(15) \begin{equation*} \mathrm{E}={\mathrm{C}}_{\mathrm{Th}}\cdot \mathrm{B}\cdot \mathrm{t}\cdot{\mathrm{d}}^{\hbox{'}}. \end{equation*}

*B* is the breathing rate and *d’* denotes the dose coefficient for inhalation. It should be noted that the progeny of ^224^Ra is considered via the following PAEC measurement.

Comparing the calculated dose (~210 ${\mu}$Sv) with the background from natural radiation sources, stated in the UNSCEAR report from 2000 ^(12)^ with 5.8 ${\mu}$Sv for the uranium and thorium series, justifies neglecting the background.

The breathing rate *B* = $28.8\ {\mathrm{m}}^3{\mathrm{d}}^{-1}$ for occupational exposure, taken from the UNSCEAR report from 2000^(12)^, exceeds the one for members of the public because workers perform physical activity during the exposure. We assume *t* = $2000 \,$h of work per year and apply the dose coefficient *d’* for ^232^Th bound to aerosols of 5 ${\mu}$m median diameter valid for occupational exposure^(26)^.

The model for the exposure because of the obtained annual PAEC (*J*) levels is shown as follows:


(16) \begin{equation*} \mathrm{E} =\mathrm{J} \cdot \mathrm{t}\cdot \frac{1\ \mathrm{WLM}}{170\ \mathrm{WLh}}\cdot \mathrm{u}. \end{equation*}

First, we convert the unit of the PAEC from working level (WL) to working level month (WLM) by using the annual working hours (*t*), guided by the Bundesamt für Strahlenschutz^(34)^. Afterwards, multiplying with the dose conversion factor (*u*) results in the annual effective dose. In contrast to the original study by Haridasan *et al*.^(33)^, an updated dose conversion factor from WLM to the effective dose of $5\ \mathrm{mSv}\ \mathrm{WL}{\mathrm{M}}^{-1}$ instead of $1.67\ \mathrm{mSv}\ \mathrm{WL}{\mathrm{M}}^{-1}$ is used, based on the recommendation of the German commission on radiological protection^(35)^, which leads to an overall higher contribution by thoron progeny.

No information on whether a background was considered in Bellamy^(32)^ is available. Because of the comparatively low ratio of background to signal for airborne ^232^Th at this facility, we assume that no correction is needed for the measured PAEC. Summing up both contributions regarding inhalation yields an annual dose of 2.62 mSv for employees of the processing facility, out of which 2.41 mSv can be attributed to thoron progeny.

For the external gamma exposure, the following model is used: 


(17) \begin{equation*} \mathrm{E}=\left(\dot{\mathrm D}-{\dot{\mathrm D}}^{\ast}\right)\cdot \mathrm{t}\cdot \mathrm{d}. \end{equation*}

$\dot{D}$ denotes the dose rate, and ${\dot{D}}^{\ast }$ denotes the dose rate because of natural background measured outside of the premises of the facility. *d* is the conversion factor given as $0.7\ \mathrm{Sv}\ \mathrm{G}{\mathrm{y}}^{-1}$^(12)^. *t* refers to the exposure time. All used parameters are compiled in Table 10.

**Table 10:** Used parameters for the exposure because of the processing of rare-earths. ^a^ indicates a particular value of the raw data.

Symbol	Name	Value	Unit
E	Additional annual effective dose		mSv
B	Breathing rate	28.8	m^3^ d^−1^
t	Annual working hours	2000	h
${\mathrm C}_{\mathrm{Th}}$	Equivalent activity of ^232^Th concentration in air	3^a^	mBq m^−3^
d’	Dose coefficient of ^232^Th (medium, 5 ${\mu} \mathrm{m}$)	29	${\mu}$ Sv Bq^−1^
J	Potential alpha energy concentration	41^a^	mWL
u	Dose conversion factor from WLM to effective dose	5	mSv WLM^−1^
m	Needed mass of neodymium per 3 MW turbine	560	kg
$\dot{\mathrm D}$	External gamma exposure rate	0.5^a^	${\mu} \mathrm{Gy}\ {\mathrm{h}}^{-1}$
${\dot{\mathrm D}}^{\ast }$	Background external gamma exposure rate	0.15	${\mu} \mathrm{Gy}\ {\mathrm{h}}^{-1}$
d	Conversion factor from dose rate to effective dose rate	0.7	Sv Gy^−1^
M	Total mass of produced rare-earth metals	3 600 000	kg
N	Number of exposed persons working in processing plant	380	

Neither name of the company nor information on the annual production and the number of employees of the processing plant was given in the original study^(33)^. However, the authors acknowledged the “Rare Earths Division Aluva” for allowing them to perform measurements at their site. According to the company’s website, this particular facility produces 3.6 million kg of rare-earths per year^(36)^. The detailed information agrees well with the examined ores and the location stated in the paper. No information on the number of employers on this specific site is available. However, in their annual report 17/18^(37)^ the company lists a total of 1521 employees. Four running facilities are mentioned on the website; therefore, we assume one-fourth of the workforce is exposed.

The weighted annual effective dose and the collective dose because of external exposure in the processing plant are calculated as follows: 


(18) \begin{equation*} {\displaystyle \begin{array}{l} {\mathrm{E}}^{\ast} =0.49\cdot{10}^{-3}\ \mathrm{Sv}\cdot \displaystyle\frac{560\ \mathrm{kg}}{3\ 600\ 000\ \mathrm{kg}}\\[10pt] \quad \ =7.62\cdot{10}^{-8}\ \mathrm{Sv} \end{array}\quad}\quad \end{equation*}

(19) \begin{equation*} {\displaystyle \begin{array}{l} {\mathrm{S}}=7.62\cdot{10}^{-8}\ \mathrm{Sv}\cdot 380 \\[4pt] =2.90\cdot{10}^{-5}\ \mathrm{manSv}.\qquad \quad \end{array}\quad}\quad \end{equation*}

## Discussion and overall evaluation

The overall collective dose per GW per year is calculated by summing up the collective doses of the individual sources, shown in Table 11. By using the parameters of a typical 3 MW wind turbine, discussed earlier, we calculate an overall collective dose per GW per year of 1.15 manSv/(GW a). Including uncertainties, the value ranges from 1.92e-1 manSv/(GW a) to 3.15 manSv/(GW a). 


(20) \begin{equation*} {\displaystyle \begin{array}{l} {\mathrm S}_{\mathrm a}=\displaystyle\frac{1.86\cdot{10}^{-2}\ \mathrm{manSv}}{3\cdot{10}^{-3}\ \mathrm{GW}\cdot 0.24\cdot 22.5\ \mathrm{a}}\\[10pt] \quad \ =1.15\ \mathrm{manSv}\ {\mathrm{GW}}^{-1}{\mathrm{a}}^{-1} \end{array}\quad}\quad \end{equation*}

Summing up the assumed number of exposed persons for every source in this study results in ~3.3 million exposed persons, out of which about 193 000 can be attributed to ferrous metals. It has to be considered that the evaluated sources cover similar geographical areas but different exposure pathways. So the densely populated Baotou City (1.5 million) is included once for every exposure pathway.

**Table 11:** Overview of examined sources, E for external exposure, I for inhalation, o for occupational dose and p for dose of the public. FM indicates sources attributed to ferrous metal production processes, whereas N indicates sources assigned to neodymium production processes.

Name	Process	Path of exposure	Exposed group	Effective dose (mSv)	Weighted effective dose (mSv)	Collective dose (manSv)
Tailing iron	FM	E	o	4.49e−3	1.08e−9	1.08e−7
Surrounding of steel plant	FM	I	p	1.20e−2	3.28e−10	6.30e−5
Inhalation of dust at steel plant	FM	I	o	2.84e−2	6.85e−9	4.80e−6
Inhalation Bayan Obo	N	I	p	1.14e−0	1.16e−8	2.67e−4
Inhalation Baotou City	N	I	p	1.11e−0	1.13e−8	1.70e−2
Bayan Obo tailing	N	E	o	3.57e−1	3.63e−9	3.63e−7
Bayan Obo work, tailing	N	E	o	1.37e−1	1.39e−9	8.23e−6
Bayan Obo living area	N	E	p	3.63e−1	3.70e−9	6.29e−5
Baotou City tailing	N	E	o	3.05e−1	3.11e−9	1.86e−6
Baotou City work, tailing	N	E	o	1.50e−2	1.53e−10	6.02e−6
Baotou City living area	N	E	p	6.90e−2	7.03e−10	1.03e−3
Processing plant	N	E	o	4.90e−1	7.62e−8	2.90e−5
Processing plant	N	I	o	2.62e−0	4.08e−7	1.55e−4
Total						1.86e−2

**Table 12:** Comparison of different energy sources based on the UNSCEAR report from 2016^(3)^.

Dose (manSv/(GW a))	Power by solar	Power by wind	Power by coal, modern	Power by coal, old	Nuclear power	Power by wind, calc
Collective dose, materials^a^	8.00e−1^d^	1.00e−1	1.00e−2	1.00e−2	2.00e−1	1.27e−2
Collective dose, public^b^	/^e^	/^e^	7.00e−1	1.40e−0	4.30e−1	1.14e−0
Subtotal	8.00e−1	1.00e−1	7.10e−1	1.41e−0	4.50e−1	1.15e−0
Collective dose, employees^c^	/^f^	/^f^	1.10e+1	1.10e+1	4.50e−0	/^g^
Total	8.00e−1	1.00e−1	1.17e+1	1.24e+1	4.95e−0	1.15e−0

a Occupational dose only because of mining and processing of materials.

b All shares of collective dose that contribute to an exposure of the public.

c Occupational dose because of operation of examined power plant.

d In the UNSCEAR 2016 report, only radiological relevant sources are examined, no information given on why it does not affect the public.

e Raw earth metals are considered.

f No data given.

g Assumption: no exposure during operation, decommissioning.

Table 11 lists the mean annual effective dose, before weighting with the needed materials. Because of the missing weighting, these doses cannot entirely be attributed to the materials needed for wind turbines. Having that in mind, the effective dose to citizens of Bayan Obo and Baotou City exceeds 1 mSv, which is the recommendation of ICRP for members of the public^(38)^. Combining the exposure pathways of inhalation and external radiation results in 1.5 mSv for the region around the mine and 1.8 mSv around the smelting area. Those values are not even overconservative, since an additional dose might originate from the ingestion pathway that was not considered in the present work because of very large uncertainties in dietary habits and probably low rate of self-supply.

Furthermore, the calculated doses in this paper refer to adults. However, exposure to the population does include different age groups. Therefore, a further development of the model should consider the exposure of age groups different from adults, too.

Our calculated value for occupational dose because of mining and processing (1.27e−2 manSv/GW/a) is lower than proposed by UNSCEAR for wind energy (0.1 manSv/GW/a) in their report from 2016^(3)^, see Table 12. The biggest share of our calculated dose is because of exposure of the public (1.14 manSv/GW/a), which is not given for wind energy in the UNSCEAR report. Therefore, it is not reasonable to compare the calculated collective dose per GW per year that includes dose to workers and public with the given value of UNSCEAR for power by wind that includes only workers.

To rank the obtained values within the energy sector, a comparison with the values from UNSCEAR 2016 for other technologies is done, listed in Table 12. When the dose for employees during operation is deducted, the calculated value is the second highest collective dose right after the collective dose of coal power plants. The ranking changes when we include the dose to operators too. The calculated collective dose for wind energy amounts to one-tenth of the collective dose because of the operation of coal plants and 23% of the collective dose because of nuclear power; however, it is a factor of 1.4 higher than the proposed value of photovoltaic electricity generation, which still lies within the calculated uncertainties.

Looking at the different collective dose contributions, our study shows that the radiation exposure primarily originates from densely populated urban areas located close to the processing facilities. Especially elevated values in Baotou City or Bayan Obo result in a high contribution to the collective dose. Consequently, there is a strong benefit of implementing new or improving established radiation protection measures for the public (and workers).

Coming back to the question raised in Introduction: countries worldwide that operate wind turbines cause radiation exposure to China (or other countries mining and processing rare-earth minerals). This effect of shifting exposures to other countries can be called ‘virtual export of doses’. Wind turbines to generate power, in most cases, are not located in the country that mines the raw materials and carries out the ‘dirty’ steps of the processing. As a consequence, the benefits and risks (e.g. dose) are geographically decoupled for wind turbines. The location where the wind turbines are operated does not fully bear the radiological risks of wind technology. This finding should be considered in communication about wind energy and furthermore increase awareness for radiation protection measures in countries mining and processing rare metals.
